# Socioeconomic status of practice location and Australian GP registrars’ training: a cross-sectional analysis

**DOI:** 10.1186/s12909-022-03359-x

**Published:** 2022-04-15

**Authors:** Dominica Moad, Amanda Tapley, Alison Fielding, Mieke L. van Driel, Elizabeth G. Holliday, Jean I. Ball, Andrew R. Davey, Kristen FitzGerald, Neil A. Spike, Parker Magin

**Affiliations:** 1 The University of Newcastle, School of Medicine and Public Health, Callaghan, NSW Australia; 2 GP Synergy, Regional Training Organisation, NSW & ACT Research and Evaluation Unit, Newcastle, NSW Australia; 3 The University of Queensland Faculty of Medicine, Primary Care Clinical Unit, Brisbane, QLD Australia; 4 Hunter Medical Research Institute, Clinical Research Design, IT and Statistical Support Unit (CReDITSS), New Lambton, NSW Australia; 5 University of Tasmania, School of Medicine, Hobart, TAS Australia; 6 General Practice Training Tasmania (GPTT), Regional Training Organisation, Hobart, TAS Australia; 7 Eastern Victoria General Practice Training (EVGPT), 15 Cato Street, Hawthorn, VIC 3122 Australia; 8 Monash University, School of Rural Health, Churchill, VIC 3842 Australia; 9 Department of General Practice, The University of Melbourne, Carlton, VIC 3053 Australia; 10 University of Newcastle, School of Medicine and Public Health, University Drive, Callaghan, NSW 2308 Australia

**Keywords:** Socioeconomic status, General Practice, Vocational Training

## Abstract

**Background:**

Socioeconomic status (SES) is a major determinant of health. In Australia, areas of socioeconomic disadvantage are characterised by complex health needs and inequity in primary health care provision. General Practice (GP) registrars play an important role in addressing workforce needs, including equitable health care provision in areas of greater socioeconomic disadvantage.

We aimed to characterize GP registrars’ practice location by level of socioeconomic disadvantage, and establish associations (of registrar, practice, patient characteristics, and registrars’ clinical behaviours) with GP registrars training being undertaken in areas of greater socioeconomic disadvantage.

**Methods:**

A cross-sectional analysis from the Registrars’ Clinical Encounters in Training (ReCEnT) study. ReCEnT is an ongoing, multi-centre, cohort study that documents 60 consecutive consultations by each GP registrar once in each of their three six-monthly training terms. The outcome factor was the practice location’s level of socioeconomic disadvantage, defined using the Index of Relative Socio-economic Disadvantage (SEIFA-IRSD). The odds of being in the lowest quintile was compared to the other four quintiles. Independent variables related to the registrar, patient, practice, and consultation.

**Results:**

A total of 1,736 registrars contributed 241,945 consultations. Significant associations of training being in areas of most disadvantage included: the registrar being full-time, being in training term 1, being in the rural training pathway; patients being Aboriginal or Torres Strait Islander, or from a non-English-speaking background; and measures of continuity of care.

**Conclusions:**

Training in areas of greater social disadvantage, as well as addressing community need, may provide GP registrars with richer learning opportunities.

## Background

Socioeconomic status (SES) is a major determinant of health, and of central importance to the work of health care providers, including general practitioners (GPs, family physicians) [1, 2]. At the individual and area of residence level, greater socioeconomic disadvantage is associated with a disproportionate burden of disease, with higher rates of illness across all categories, particularly chronic diseases and multi-morbidity, and within a wider context of the concomitants of lower SES, including under- or unemployment, insecure housing, and poor social supports [3–5]. There are strong associations of residing in an area of greater socioeconomic disadvantage and disease risk factors, including smoking and poor nutrition, as well as lower uptake of preventative care including immunisations and health-screening [4–7].

An adequate supply of primary care physicians attenuates disparities in health across socioeconomic status [2]. But people in areas of greater socioeconomic disadvantage have increased difficulty accessing primary healthcare, including longer wait times and shorter consultation times, resulting in lower rates of patient enablement and patient satisfaction [3, 8–12]. This represents a manifestation of the ‘inverse care law’, where workforce shortages and maldistribution see those with the highest need of healthcare receiving the least care [9, 13].

Thus, addressing SES-related health inequities relies on an ongoing workforce of adequately educated and trained general practitioners (GPs). The training period for GP registrars (specialist vocational trainees in general practice) provides an opportunity to influence their future work practices as GPs, and can assist in preparing GPs to respond to the medical, psychological and social needs of the most socioeconomically disadvantaged within Australia’s evolving primary health care system.

Clinical experience is fundamental in the adequate preparation of GP registrars for the complexities and challenges of future independent practice. Structural changes in junior hospital doctor clinical experience can limit pre-vocational exposure to factors critical to care of disadvantaged populations, including the comprehensive management of patients with chronic disease and multi-morbidity. This makes it more important that registrars gain adequate exposure during vocational training [14].

It is therefore plausible that GP registrars training in areas of lower SES may benefit from a richer training experience, with increased exposure to higher levels of multimorbidity and more complex medical and psychosocial patient presentations. GPs practising in areas of socioeconomic disadvantage encounter higher rates of complex multi-morbidity and chronic disease, and may have a greater engagement in promoting preventative health care (through screening for biological and behavioural influences on health) [3]. However, the potential educational benefits from training in areas of socioeconomic disadvantage have not been well-established or explored.

In addition to preparing registrars clinically, it is argued that government-funded GP training organisations bear a social obligation to acknowledge, and redress the inequality in healthcare across socioeconomic areas [15, 16]. Promoting training in high-needs areas provides both short- and long-term benefits [16]. By training in areas of socioeconomic disadvantage, GP registrars contribute to the current GP workforce in often-underserved areas, while obtaining a real world orientation to their social responsibilities as GPs.

The current exploratory study aimed to a) characterize GP registrars’ practice location by level of socioeconomic disadvantage, and b) to establish associations of training in areas of greater socioeconomic disadvantage; including registrar, practice, and patient characteristics, and registrars’ clinical behaviours.

## Methods

This cross-sectional analysis took place within the Registrars’ Clinical Encounters in Training (ReCEnT) study.

### ReCEnT

ReCEnT is a cohort study of individual registrars’ in-consultation clinical and educational experience. The complete methodology is described elsewhere [17]. Briefly, GP registrars collect data once at approximately the mid-point in each of their three six-month mandatory general practice training terms, capturing demographic data, diagnoses, investigations/management, and educational training aspects of 60 consecutive patient consultations. The project is an intrinsic element of registrars’ training, and is compulsory [18, 19]. Registrars may also provide informed written consent to their data being used for research purposes. From 2010 to 2015 it was conducted in Regional Training Providers (RTPs) across five of Australia’s six states and, from 2016 (after a reorganization of Australian GP vocational training), in three Regional Training Organizations (RTOs) in three Australian states and the Australian Capital Territory.

The number of registrars from participating RTPs/RTOs consenting to use of ReCEnT for research purposes determined the sample size for this study.

### Patient and Public Involvement

Patients or members of the public were not involved as participants in this study.

### Outcome factor

The outcome factor was a measure of the registrar’s practice location level of socioeconomic disadvantage. Practice location postcode was used to define the practice Socio-Economic Index for Area Relative Index of Disadvantage (SEIFA-IRSD) which we determined to be the most appropriate of the SEIFA indexes for this research question [20]. The SEIFA -IRSD summarises a range of social and economic variables of an area to provide an index of relative disadvantage. While low income is the strongest indicator of disadvantage, additional variables include employment type/unemployment, education, rent repayments, disability, internet connection, and household relationships such as single parenting, separation, and divorce [20].

All GP training practices who to have participated in ReCEnT were ranked by SEIFA-IRSD. The ranked-by-SEIFA-IRSD practices were categorized to form five quintiles, and then stratified so the SEIFA-IRSD quintile of greatest disadvantage was compared to the other four quintiles.

### Independent variables

Independent variables related to the registrar, patient, practice, consultation, and consultation outcomes.

Registrar variables included age, gender, full-time/part-time status, training term, place of medical qualification (Australia or International), training pathway, non-English speaking background, and whether the registrar had worked at the practice before.

Practice variables included practice size (number of full-time equivalent GPs, with practices with less than five GPs categorised as small), geographic location (rurality) (using practice postcode to define Australian Standard Geographical Classification-Remoteness Area, ASGC-RA) [21], training region, and bulk-billing policy (whether consultations are free to the patient).

Patient characteristics included age, gender, and whether the patient identified as Aboriginal and/or Torres Strait Islander, was from a non-English speaking background, and was a continuing patient or was new to the practice, or to the registrar.

Consultation characteristics included consultation duration, number of problems/diagnoses managed, and whether the registrar sought information or assistance during the consultation (from their supervisor/trainer, from a specialist, or from hard-copy or electronic sources), whether the problem was classified as a chronic disease [22], if any procedures were performed, and if the patient was seen by a practice nurse.

Consultation outcomes included whether any imaging or pathology tests were ordered, whether any follow-ups were arranged, if any medications were prescribed, if any referrals were made, and if the registrar generated any learning goals during the consultation.

### Statistical Analysis

This was a cross-sectional analysis. Analysis was performed on 16 rounds of data collected between 2010 and 2017. Individual regions contributed 2 to 17 rounds of data depending on when they entered the project and on continuity/discontinuity across the 2015-2016 restructure of Australian GP vocational training. The unit of analysis was the consultation.

The proportion of consultations in the lowest SEIFA-IRSD quintile was calculated with 95% Confidence Interval (CIs).

Univariate logistic regressions were undertaken to examine the relationships between the outcome factor and independent variables. Variables with a *P*-value of <0.20 were considered for inclusion in the multivariable logistic regression model. Logistic regression was used within the generalised estimating equations framework, to account for repeated measures within registrars. Once multivariable models were fitted, model reduction was assessed. Covariates not reaching p<0.20 in the multivariable model were tested for removal from the model. If the covariate’s removal did not substantively change the resulting model (defined as any covariate in the model having a change in the effect size (odds ratio) of greater than 10%), the covariate was removed from the final model.

To examine different facets of our research question, three models were built, each with ‘quintile of greatest socioeconomic disadvantage’ as the dependent variable.

To examine the associations of a consultation being conducted in the greatest disadvantage quintile (i.e., lowest SEIFA-IRSD quintile), patient, practice and registrar independent variables were included in a multivariable regression model.

To examine how consultations conducted in the area of greatest disadvantage quintile differ from other consultations, the above variables were included in a second multivariable model along with the following additional variables: consultation duration, the number of problems addressed during the consultation, if chronic conditions were managed, and if any sources of information or advice were consulted.

To examine how outcomes of consultations in areas of the quintile of greatest disadvantage compared to those of other consultations, all variables from the previous models were included in a final multivariable model along with the following additional variables: if procedures were performed, follow-up organised, and whether learning goals were generated.

The rationale for building three models was that associations of a registrar’s consultation being conducted in the lowest SEIFA-IRSD quintile practice will include patient, registrar and practice factors, but evaluation of these associations may be compromised by inclusion in the multivariable model of factors operating once the consultation is progressing. Similarly, evaluation of the content of the consultation may be compromised by the inclusion in this model of outcomes arising from the consultation.

Of the 29 covariates of interest, 24 were considered for inclusion in the multivariable model. However, ‘region’ and ‘rurality’, were subsequently removed from the model, due to high correlation of these variables with each other and with the outcome, causing instability of parameter estimates due to data sparsity and collinearity.

Variables were considered statistically significant if the *P*-value was <0.05.

Analyses were completed using Stata 13.1 (Statacorp, Texas, USA) and SAS version 9.4.

### Ethics approval

Ethics approval was from the University of Newcastle Human Research Ethic Committee, Reference H-2009-0323.

## Results

The analyses included 1,736 individual registrars (response rate 96.2%) contributing 241,945 consultations, of which 44,310 (18.3% [95% CI: 18.2-18.5]) were conducted in practices in the lowest SEIFA-IRSD quintile.

Characteristics of the registrars and their practices are shown in Table 1.

**Table 1:** Characteristics of participating registrars, including by round of data collection (registrar-round)

**Registrar characteristics (*n*=1736)**		**n (%)**
Registrar gender	Female	1114 (64.2)
Qualified as doctor in Australia	Yes	1724 (82.5)
Training Pathway	General	1277 (74.1)
**Registrar-round characteristics (*n*= 4072)**		
Registrar works full-time	Yes	3077 (77.7)
Age (years)	Mean ± SD	32.4 ± 6.1
	<=30	1825 (45.9)
	31-40	1684 (42.3)
	41-50	389 (9.8)
	51+	80 (2.0)
Training term	Term 1	1614 (39.6)
	Term 2	1469 (36.1)
	Term 3	989 (24.3)
Registrar worked at practice previously	Yes	994 (24.7)
Practice routinely bulk bills	Yes	894 (22.3)
Practice Size (No. GPs working at the practice)	Small (1-4 GPs)	1427 (36.1)
	Large (5+ GPs)	2527(63.9)
Rurality	Major city	2443 (60.2)
	Inner regional	1024 (25.2)
	Outer regional remote	594 (14.6)

Characteristics associated with training in a practice in the lowest SEIFA-ISRD quintile versus the four highest quintiles are presented in Table 2.

**Table 2:** Characteristics associated with lowest SEIFA-IRSD quintile^a^ and highest 4 SEIFA-IRSD quintiles^a^ (*n*=241,945)^b^

Variable	Class	Lowest quintile^a^ n (%)	Highest 4 quintiles^a^ n (%)	p
**Registrar Variables**				
Registrar gender	Male	18306 (41)	70561 (36)	0.064
	Female	26004 (59)	127074 (64)	
Registrar Full-Time or Part-Time	Part-time	9488 (22)	42851 (22)	<0.001
	Full-time	33876 (78)	149195 (78)	
Training term	Term 1	19348 (44)	76513 (39)	<0.001
	Term 2	15661 (35)	71614 (36)	
	Term 3	9301 (21)	49508 (25)	
Worked at practice previously	No	31894 (73)	147753 (76)	<0.001
	Yes	11996 (27)	47085 (24)	
Qualified as doctor in Australia	No	9075 (21)	33516 (17)	0.201
	Yes	34995 (79)	162779 (83)	
Pathway	General	30867 (70)	149094 (76)	0.181
	Rural	13145 (30)	47289 (24)	
Non English Speaking background	No	9368 (21)	30096 (15)	0.01
	Yes	34762 (79)	165988 (85)	
Registrar age	mean (SD)	32 (6)	32 (6)	<0.001
**Practice Variables**				
Practice size	Small	20593 (47)	64277 (34)	<0.001
	Large	22770 (53)	127297 (66)	
Region	Region 1	7196 (16)	57084 (29)	<0.001
	Region 2	5628 (13)	17844 (9)	
	Region 3	11663 (26)	18762 (9)	
	Region 4	15775 (36)	82700 (42)	
	Region 5	527 (1)	6489 (3)	
	Region 6	3521 (8)	14756 (7)	
Practice routinely bulk bills	No	26883 (62)	158480 (81)	<0.001
	Yes	16647 (38)	36595 (19)	
Rurality	Major city	23291 (53)	121805 (62)	<0.001
	Regional/remote	21019 (47)	75207 (38)	
**Patient Variables**				
Patient age group	0-14	6818 (16)	35095 (18)	<0.001
	15-34	11976 (27)	52934 (27)	
	35-64	16778 (38)	72566 (37)	
	65+	8130 (19)	34045 (17)	
Patient gender	Male	17252 (40)	75055 (39)	0.015
	Female	26077 (60)	117639 (61)	
Aboriginal and Torres Strait Islander	No	40277 (98)	183157 (99)	<0.001
	Yes	889 (2)	2517 (1)	
Non-English Speaking Background	No	35225 (85)	175804 (94)	<0.001
	Yes	6371 (15)	11115 (6)	
Patient/practice status	Existing patient	19487 (45)	77234 (40)	<0.001
	New to registrar	20838 (48)	102124 (53)	
	New to practice	2910 (7)	14067 (7)	
**Consultation Variables**				
Sought assistance (any source)	No	33095 (75)	153168 (78)	<0.001
	Yes	11215 (25)	44467 (22)	
Chronic problem	No	31631 (71)	145957 (74)	<0.001
	Yes	12679 (29)	51678 (26)	
Procedure performed	No	39529 (89)	178213 (90)	0.002
	Yes	4781 (11)	19422 (10)	
Seen by practice nurse	No	39847 (90)	177741 (91)	0.719
	Yes	4215 (10)	18504 (9)	
Consultation duration	mean (SD)	18 (9)	17 (9)	<0.001
Number of problems managed	mean (SD)	2 (1)	2 (1)	<0.001
**Consultation Outcomes Variables**				
Imaging ordered	No	39405 (89)	175527 (89)	0.646
	Yes	4905 (11)	22108 (11)	
Follow-up ordered	No	18096 (41)	89785 (45)	<0.001
	Yes	26214 (59)	107850 (55)	
Pathology ordered	No	34649 (78)	153738 (78)	0.430
	Yes	9661 (22)	43897 (22)	
Medication prescribed	No	18330 (41)	84381 (43)	0.276
	Yes	25980 (59)	113254 (57)	
Referral made	No	36744 (83)	163768 (83)	0.700
	Yes	7566 (17)	33867 (17)	
Learning goals generated	No	31167 (73)	145215 (76)	<0.001
	Yes	11494 (27)	45812 (24)	

^a^Quintile based on SEIFA-IRSD categorisation within the population of ReCEnT participating teaching practices.

^b^numbers may not add up to 241,945 due to missing data

Results of univariate and multivariable logistic regression models are presented in Table 3.

**Table 3:** Associations of registrars’ practice location socioeconomic status from univariate and multivariable logistic regression

			Univariate		Adjusted	
Factor group	Variable	Class	OR (95% CI)	p	OR (95% CI)	p
**Model i. Registrar, Practice and Patient Variables**						
**Registrar Variables**						
Non-English Speaking Background		Yes	0.42 (0.21, 0.81)	0.011	0.33 (0.14, 0.76)	0.009
Registrar Full-time or Part-time		Part-time	0.56 (0.53, 0.60)	<.001	0.48 (0.44, 0.52)	<.001
Pathway		Rural	1.48 (0.83, 2.63)	0.181	4.22 (2.06, 8.65)	<.001
Registrar age			0.65 (0.63, 0.66)	<.001	0.75 (0.72, 0.78)	<.001
Registrar gender		Female	0.61 (0.36, 1.03)	0.064	0.65 (0.34, 1.22)	0.179
Training term Referent: Term 1		Term 2	0.70 (0.67, 0.73)	<.001	0.64 (0.60, 0.68)	<.001
		Term 3	0.46 (0.44, 0.48)	<.001	0.50 (0.46, 0.54)	<.001
Worked at practice previously		Yes	0.74 (0.70, 0.78)	<.001	1.22 (1.13, 1.31)	<.001
**Practice Factors**						
Practice routinely bulk bills		Yes	13.8 (12.8, 14.7)	<.001	17.5 (16.1, 19.0)	<.001
Practice size		Small	3.75 (3.55, 3.95)	<.001	3.62 (3.38, 3.87)	<.001
**Patient factors**						
Patient Aboriginal or Torres Strait Islander		Yes	1.74 (1.48, 2.04)	<.001	1.49 (1.23, 1.80)	<.001
Patient Non-English Speaking Background		Yes	3.70 (3.48, 3.94)	<.001	2.89 (2.68, 3.13)	<.001
Patient gender		Female	0.96 (0.92, 0.99)	0.015	0.94 (0.90, 0.99)	0.015
Patient age group Referent: 15-34		0-14	0.86 (0.81, 0.91)	<.001	0.91 (0.85, 0.98)	0.011
		35-64	1.02 (0.98, 1.07)	0.350	1.10 (1.04, 1.16)	0.001
		65+	0.97 (0.92, 1.02)	0.262	1.05 (0.98, 1.12)	0.195
Patient/practice status Referent: Existing Patient		New to practice	0.86 (0.80, 0.93)	<.001	0.89 (0.81, 0.97)	0.010
		New to registrar	0.85 (0.82, 0.88)	<.001	0.89 (0.84, 0.93)	<.001
**Model ii. Registrar, practice, patient, and all consultation variables**						
**Consultation Variables**						
Chronic problem		Yes	1.12 (1.08, 1.17)	<.001	1.04 (0.99, 1.10)	0.138
Consultation duration			1.01 (1.01, 1.01)	<.001	1.00 (1.00, 1.00)	0.878
Number of problems managed			1.08 (1.05, 1.10)	<.001	1.03 (1.00, 1.07)	0.057
Procedure performed		Yes	1.09 (1.03, 1.16)	0.002	1.07 (0.99, 1.15)	0.101
Sought help any source		Yes	1.10 (1.05, 1.15)	<.001	0.99 (0.93, 1.05)	0.715
**Model iii. Registrar, practice, patient, and all consultation variables**						
**Consultation Outcome Variables**						
Learning goals generated		Yes	1.11 (1.06, 1.16)	<.001	0.99 (0.93, 1.05)	0.680

### Multivariable associations

Statistically significant (at *p*<0.05 level) registrar-level multivariable associations of conducting a consultation in the lowest SEIFA-IRSD quintile practices included: the registrar working full-time (OR 0.48 [95% CI: 0.44, 0.52] for part-time work), being on the rural training pathway (OR 4.22 [95% CI: 2.06-8.65]), and to have worked at the practice before (OR 1.22 [95% CI: 1.13, 1.31]). Younger (OR 0.75 [95% CI: 0.72- 0.78] for each year) and less experienced (ORs 0.64 [95% CI: 0.60-0.68] and 0.50 [95% CI: 0.46-0.54] for Terms 2 and 3, respectively, compared to Term 1) registrars were more likely to undertake training in practices of lowest SEIFA-IRSD quintile.

Statistically significant practice-level factors included small practice size (OR 3.62 [95% CI: 3.38- 3.87]) and routinely bulk-billing (OR 17.5 [95% CI: 16.1-19.0]). Significant patient-level associations included: the patient being of Aboriginal and Torres Strait Island background (OR 1.49 [95% CI: 1.23- 1.80]), of non-English speaking background (OR 2.89 [95% CI: 2.68-3.13]), having seen the registrar previously (OR 0.89 [95% CI: 0.84, 0.93] for being new to the registrar and OR 0.89 [95% CI: 0.81-0.97] for being new to the practice).

There were no significant multivariable consultation-level or consultation-outcome associations.

## Discussion

### Main Findings and comparison with existing literature

While existing research into established GPs and GP registrars often examines socioeconomic status as a study variable, few studies focus on SES as the outcome factor. This study is therefore unique in highlighting key considerations for GP registrars training in practices in areas of socioeconomic disadvantage.

In this study, registrars who undertook training in areas of greater socioeconomic disadvantage had greater exposure to patient populations known to have a greater burden of chronic disease, multi-morbidity, and complex social needs. This included patients of Aboriginal and/or Torres Strait Islander background, and patients from a non-English-speaking background [5]. This is consistent with patient demographics of areas of greater socioeconomic disadvantage in Australia [23].

GP registrar’s continuity of care was also a theme of the results, with patients in more disadvantaged areas being more likely to have seen the registrar previously. The patient was also less likely to be new to the practice. And registrars in disadvantaged area practices were more likely to have worked at the practice previously. This cluster of findings suggests continuity of care. Maintaining a relationship with a GP is an essential element to patient engagement and satisfaction [12], and is also recognised as of significant benefit for the registrar in preparing them for independent practice [24].

Registrars were also more likely to be on the rural pathway, which we have found previously to provide a more diverse clinical experience [25], and there was some evidence (*p*=0.057) for an association with more problems seen per encounter than in areas of higher SES. These findings, along with the opportunity to treat patients from higher-needs groups and increased continuity of care, support the potential for a clinically richer training experience for registrars at practices in of greater socioeconomic disadvantage.

We also found that GP registrars in their first training term were significantly more likely to work in areas of greater disadvantage than those in later terms. This suggests an educational ‘immersion’ in the rich training experience that these practices may offer. This may be similar to registrars being exposed to the rich learning environment of rural practice at the beginning of their training [25].

### Strengths and limitations

A strength of this study is the generalisability of results. The ReCEnT study covers all categories of rurality and includes a comprehensive mix of SES areas. The large sample size and a high response rate [26], as well as a comprehensive list of relevant independent variables making for a fine-grained exploration of associations of practice SES are strengths.

Due to the cross-sectional nature of this study, however, we can only demonstrate associations, not causation.

A possible limitation of this study is the use of practice, as opposed to patients’, geographic level of disadvantage. However, while practices themselves may see a mix of patients from all SES backgrounds, the focus of this study was to highlight the experiences of registrars within these practices overall, rather than with any individual patient.

A further limitation is that we have data only on the content of individual consultations. We do not have data on past medical or social history, or medicine regimens.

### Implications for policy and practice

There were associations identified in this study that indicate a richer training experience for GP registrars who train in practices located in areas of greater socioeconomic disadvantage. These include associations with working with patients from groups with clinical complexity, and with markers of greater continuity of care. This may contribute to registrars’ learning, as well as helping meet the current health care needs of disadvantaged areas (noting that registrars comprise 13% of Australia’s general practice workforce (by headcount) [27, 28]). An implication is that registrars should be strongly encouraged to train in lower SES-areas.

It might be thought that early exposure to the richness and complexity of medicine in disadvantaged areas may, in itself, encourage registrars to continue to work in these areas, including post-Fellowship. Our findings, however, of an association of later training term with less disadvantaged practice setting may suggest that registrars may move away from more disadvantaged practices during training (though our cross-sectional study cannot establish temporal patterns in registrars’ practice location). If this is so, it may be a concern that this trend could continue into registrars’ post-Fellowship choices of practice location. This may suggest attempts to address areas of high need, such as low socioeconomic disadvantage via GP vocational training, may be limited in rebalancing health equity and workforce issues beyond the immediate effect of vocational training time.

An additional consideration is that while we have found evidence of training in lower-SES areas providing a rich educational environment, this may also represent a clinically challenging environment (especially the challenges of complex multimorbid disease in socially complex contexts). That we have found that registrar experience in lower-SES areas is ‘front-loaded’ earlier in training may have implications for vocational GP training. Within the apprenticeship-like model of Australian GP vocational training, supervisor in-practice oversight of registrars’ learning and practice is concentrated to greater support early in training. Given that structural approaches to redress the ratio of first-term to later-term registrars in disadvantaged practices may be difficult to implement, our findings suggest that front-loading of supervisory support could be even further resourced in disadvantaged areas.

### Implications for future research

Future research is required to understand in greater detail the experiences of registrars in areas of greater socioeconomic disadvantage, and what influence this has on their future placement and practice location choices. Careful consideration needs to be given to less experienced registrars training in areas of socioeconomic disadvantage, and what supports may be required if this pattern continues.

Further research of this area would assist in understanding the experiences of those working in disadvantaged areas, as well as the impact practice location SES has on the learning outcomes for GP registrars.

## Conclusion

Our findings suggest that GP registrars training in areas of greater socioeconomic disadvantage are exposed to a broader range of clinical and educational experiences and learning opportunities. Registrars should consider undertaking training in these areas to take advantage of the range of these experiences. The continued support of registrars working in these areas, and the encouragement of more senior registrars to work there, also has the potential to assist in addressing health inequity experienced within these communities.

## Data Availability

The datasets analysed during the current study is not available due to the determination of the Human Research Ethics Committee.
